# Glycated High-Density Lipoproteins Reduce Endothelial Phenotypic Expression of Monocyte-Derived Multipotential Cells in Early Type 2 Diabetes

**DOI:** 10.3390/metabo16030194

**Published:** 2026-03-15

**Authors:** Felipe Massó-Rojas, Luis Felipe Montaño-Estrada, Araceli Páez-Arenas, Juan Gabriel Juárez-Rojas, Aida Medina-Urrutia, Rafael Nambo-Venegas, Emma Rodríguez-Maldonado, Esteban Jorge-Galarza

**Affiliations:** 1 Laboratory of Translational Medicine, UNAM-INC Research Unit, National Institute of Cardiology Ignacio Chávez, Mexico City 14080, Mexico; felipe.masso@cardiologia.org.mx (F.M.-R.); araceli.paez@cardiologia.org.mx (A.P.-A.); emma.rodriguez@cardiologia.org.mx (E.R.-M.); 2 Inmunobiology Laboratory, Department of Cellular and Tissue Biology, Faculty of Medicine, Universidad Nacional Autónoma de Mexico (UNAM), Mexico City 04510, Mexico; lfmontmx@unam.mx; 3 Pharmacology Department, National Institute of Cardiology Ignacio Chávez, Mexico City 14080, Mexico; gabriel.juarez@cardiologia.org.mx (J.G.J.-R.); aida.medina@cardiologia.org.mx (A.M.-U.); 4 Laboratory of Chronic Diseases Biochemistry, National Genomics Medicine Institute (INMEGEN), Mexico City 14080, Mexico; lgenv@inmegen.edu.mx; 5 Posgrado en Ciencias Biológicas, Unidad de Posgrado, Universidad Nacional Autónoma de México (UNAM), Mexico City 04510, Mexico; 6 Department of Outpatient Care, National Institute of Cardiology Ignacio Chávez, Mexico City 14080, Mexico

**Keywords:** newly diagnosed type 2 diabetes, high-density lipoproteins, endothelial progenitor cells, advanced glycation products, high-density lipoprotein size

## Abstract

**Background**: High-density lipoproteins (HDL) exert protective effects on the endothelium, which are impaired in type 2 diabetes (T2D). Although monocyte-derived multipotential cells (MOMCs) can be differentiated into the endothelial lineage, it remains unclear whether HDL glycation, size, and composition could affect MOMCs differentiation. **Methods**: Twenty normoglycemic (49 years, 35% male), 20 prediabetic (52 years, 35% male), and 20 newly diagnosed T2D participants (51 years, 50% male) were recruited. HDL were isolated from each study group. The size, composition, and early, intermediate, or advanced glycation products of HDL were determined. CD14+ MOMCs were isolated from healthy volunteers and incubated with HDL from each group. Endothelial phenotypic expression was assessed by CD14^+^/KDR^+^ expression. **Results:** Compared with normoglycemic and prediabetic individuals, T2D patients had higher concentrations of early (4.4, 4.6, vs. 5.2 µmol/mg of protein, respectively; *p* = 0.049) and advanced (7.7, 8.7, vs. 14.3 µg-BSA-AGEs/mg of protein, respectively; *p* < 0.02) glycation products in HDL. HDL composition was similar among groups. The CD14+/KDR+ expression in MOMCs incubated with HDL from T2D patients was lower than that observed in prediabetes and normoglycemic individuals (46% vs. 52% and 61%, respectively; *p* = 0.002). Advanced glycation end products in HDL inversely correlated with CD14+/KDR+ cells (r = −0.418, *p* = 0.002), adjusting for other HDL characteristics. **Conclusions**: In T2D patients, increased HDL-advanced glycation impairs the endothelial phenotypic expression of MOMCs, independently of other HDL characteristics. Since advanced glycation leads to greater biological damage, these findings highlight the importance of preserving HDL integrity in T2D patients to support endothelial repair and potentially delay vascular complications.

## 1. Introduction

Type 2 diabetes (T2D) is characterized by chronic hyperglycemia, which promotes the formation and accumulation of non-enzymatic glycation adducts. These compounds, generated by the spontaneous reaction of glucose with arginine and lysine residues, contribute to vascular dysfunction, inflammation, and atherogenesis [1,2]. The spectrum of glycation adducts includes early glycation products (fructosamines), intermediate products (carbonylated proteins), and advanced glycation end products (AGEs). Among these, AGEs are particularly relevant due to their chemical stability and their long-term detrimental effects on organs and tissues [3].

In healthy conditions, the insults to the endothelial cells are resolved by differentiation of endothelial colony-forming cells (ECFC) and myeloid angiogenic cells (MAC) [4], which are of hematopoietic nature [5]. Monocyte-derived multipotential cells (MOMCs) are a population of MAC progenitors with fibroblast-like morphology characterized by CD14 expression [6,7]. These progenitor cells engage in vascular restoration due to their plasticity that allows them to differentiate into an endothelial-like phenotype while retaining monocytic features [8,9]. MOMCs can be considered as a rapid response system against vascular insults, easily incorporated into the injury site. Compared to EPC, which has low abundance in peripheral blood (0.01–0.0001% of PBMCs), MOMCs are significantly more abundant (5–10%) [10,11]. Therefore, this subpopulation could play a more relevant role in vascular restoration.

Previous studies have reported that high-density lipoproteins (HDL) play a relevant role in restoring vascular damage, promoting angiogenesis [12,13], or improving impaired angiogenesis [14]. HDL are heterogeneous macromolecules in shape, size, and chemical composition [15]. These properties mediate their antiatherogenic and vasoprotective functions [14]. However, continuous exposure of HDL to hyperglycemia also induces the formation of a wide range of glycation adducts, which impair the beneficial antiatherogenic properties of these lipoproteins and result in an increased risk of vascular damage [16,17,18]. In T2D, endothelial dysfunction is also a common abnormality [19], promoting the development of vascular complications such as diabetic cardiomyopathy [20]. Nevertheless, HDL from T2D patients disturbs the differentiation of ECFC [21], impairing their participation in the repair of the injured carotid artery in nude mice [22]. Furthermore, low HDL cholesterol levels (HDL-C) were associated with reduced MAC viability and adhesiveness [23]. It has been suggested that this harmful effect of HDL in T2D could be attributed to abnormal HDL composition, size, or glycation status [24]. However, the role of glycated HDL and its macromolecular composition on MOMC differentiation has been insufficiently explored. Therefore, this study aims to explore whether different types of glycation products in HDL (early, intermediate, or advanced) affect MOMCs differentiation into an endothelial-like cell phenotype. Additionally, it examines whether the size and macromolecular composition of these lipoproteins from patients with prediabetes and newly diagnosed T2D participate in this process. We hypothesized that HDL glycation would have a greater effect on the CD14+/KDR+ phenotypic expression of MOMCs compared to their macromolecular composition or size.

## 2. Materials and Method

### 2.1. Study Design and Population

The study design and methods have been previously reported [25]. The sample included 60 individuals from the control group of the Genetics of Atherosclerotic Disease study and employees from the Instituto Nacional de Cardiología Ignacio Chávez in Mexico City, Mexico. Participants met the following inclusion criteria: men and women aged 40 to 60 years, with a previous fasting plasma glucose measurement, body mass index < 33 kg/m^2^, and no personal history or diagnosis of cardiovascular, liver, kidney, or thyroid disease. Individuals under hypolipidemic and antihypertensive treatment were excluded. Participants were categorized into three groups following the American Diabetes Association criteria [26]: (1) normoglycemic group, consisting of twenty subjects defined as fasting glucose < 100 mg/dL, hemoglobin A1C (HbA1C) < 5.7% or glucose < 140 mg/dL 2 h after the oral glucose tolerance test (OGTT, 74 g oral glucose intake); (2) prediabetic group, composed of twenty individuals diagnosed by one of the following: fasting glucose 100–125 mg/dL, HbA1C 5.7–6.4%, or glucose 140–199 mg/dL, 2 h after the OGTT, and (3) newly diagnosed type 2 diabetes patients, with less than three years since diagnosis, fasting glucose > 125 mg/dL, HbA1C ≥ 6.5% or glucose ≥ 200 mg/dL 2 h after the OGTT. Exclusion criteria included: T2D patients with more than 4 years of duration, participants with aspartate aminotransferase (AST) levels greater than 126 IU/L, alanine aminotransferase (ALT) levels greater than 123 IU/L, triglyceride levels greater than 600 mg/dL, or high-sensitive C-reactive protein (hs-CRP) levels greater than 10 mg/L. Seven participants with T2D were receiving metformin treatment, and two of them were also being treated with DPP-4 inhibitors. To minimize potential bias related to medication, all T2D patients discontinued their treatments 72 h before their inclusion in the study, under strict medical supervision. After participating in the study, the patients resumed their medications.

### 2.2. Biochemical Analyses

After a 12 h overnight fast, 20 mL EDTA blood samples were drawn and centrifuged within 15 min of collection. Plasma was separated, aliquoted, and immediately analyzed or frozen at −80 °C until analysis. Plasma glucose, total cholesterol, triglycerides, HDL-C, HbA1C, apolipoprotein A1, apolipoprotein B-100, hs-CRP, ALT, and AST were measured using standardized procedures with an automated analyzer (Roche Diagnostics GmbH, Mannheim, Germany). Inter-assay coefficients of variation were less than 6% for all assays. Low-density lipoprotein cholesterol (LDL-C) was estimated by the Delong et al. formula [27]. The glomerular filtration rate (eGFR) was computed using the Chronic Kidney Disease Epidemiology Collaboration algorithm.

### 2.3. HDL Characterization

HDL of each participant were isolated from plasma by sequential ultracentrifugation. The apoB-containing lipoproteins (very low-density lipoproteins and low-density lipoproteins) were first removed by adjusting plasma to a density of 1.063 g/mL using a potassium bromide solution with EDTA at a concentration of 0.5 g/L, followed by ultracentrifugation at 110,000 RPM in a TL-110 fixed-angle rotor (Beckman Coulter, Brea, CA, USA) for 2.5 h. The top fraction was discarded, and the infranatant containing HDL was then adjusted to a density of 1.21 g/mL and centrifuged at 110,000 RPM for 3.5 h. This final step was repeated, after which the HDL-containing fraction was extensively dialyzed against phosphate-buffered saline (PBS; 10 mM, pH 7.4) at 4 °C [28]. The HDL macromolecular composition was determined by measuring the protein concentration by the Lowry method, and lipid content (triglycerides, total cholesterol, esterified cholesterol, and phospholipids) was determined using colorimetric reagents from Wako Diagnostics (FujiFilm Wako Diagnostics, Mountain View, CA, USA). The macromolecular composition of HDL was expressed as a normalized percentage of the total mass. The size and subpopulations of the HDL were determined by 4–30% gradient polyacrylamide gel electrophoresis under non-denaturing conditions, followed by densitometric analysis of the gel to estimate the average particle size of HDL and subclasses HDL2b, HDL2a, HDL3a, HDL3b, and HDL3c [29]. A control sample was processed in each assay to compute the inter-assay coefficient of variation; for these determinations, it was 5.1%.

Glycation products were measured in the total isolated HDL fraction. The early glycation product (fructosamine) was measured by enzymatic colorimetric methods in an automated analyzer (Cobas Integra Fructosamine, Roche Diagnostics, Mannheim, Germany). The intermediate glycation products were quantified by incubating HDL with 2,4-dinitro-phenylhydrazine in 2.5 N HCl to identify dicarbonyl groups, at 370 nm in a Biotek Synergy H1 microplate spectrophotometer (Biotek Instruments, Winooski, VT, USA), as previously reported [30]. The AGEs were quantified using the OxiSelect commercial competitive ELISA kit (STA-817, Cell Biolabs, San Diego, CA, USA), identifying the carboxymethyl lysine and pentosidine species. The kit includes a standard of glycated bovine serum albumin (BSA). Therefore, we expressed AGEs as μg BSA-AGE per mg of protein in HDL. A control sample was processed in each assay to compute the inter-assay coefficient of variation; for these determinations, it was 17% for fructosamine, 11.8% for carbonylated proteins, and 7.4% for AGEs determinations.

### 2.4. MOMCs Isolation

MOMCs were obtained from 50 mL of peripheral blood by isolating the peripheral blood mononuclear cells (PBMCs) fraction through density gradient centrifugation using Histopaque-1077 (Sigma-Aldrich, St. Louis, MO, USA). For each experiment, PBMCs were obtained from a single blood bank donor to avoid unintended immune activation associated with sample mixing. PBMCs were seeded on a fibronectin-coated plate with RPMI-1640 with medium supplemented with L-glutamine and 10% fetal bovine serum (SFB, Sigma-Aldrich, St. Louis, MO, USA), 2 mM L-glutamine, 50 U/mL penicillin, and 50 µg/mL streptomycin, at a density of 2 × 10^6^ cells and cultured at 37 °C in a 5% CO_2_ atmosphere for 24 h. Non-adherent cells were discarded, and adherent cells (fresh monocytes) were used as control cells or to induce MOMCs phenotypic expression, as previously reported [11]. Briefly, fresh monocytes were cultured with endothelial cell growth basal medium-2 (EBM2) supplemented with 5% fetal bovine serum, vascular endothelial grown factor (VEGF), basic fibroblast growth factor, epidermal growth factor, insulin-like growth factor-1, and ascorbic acid (Lonza Pharma, Basel, Switzerland) at 37 °C in a 5% CO_2_ atmosphere, until confluence. Fresh culture medium was added every three days until the cells were harvested for flow cytometry analysis.

### 2.5. MOMCs Endothelial Marker Expression

MOMCs obtained from endothelial-like differentiation induction of fresh monocytes were incubated with 50 µg/mL of HDL isolated from individuals of each group, using non-supplemented RPMI-1640 for 45 min in a 5% CO_2_ atmosphere at 37 °C, followed by extensive washing. The cells were washed with 10 mM PBS before being seeded in a supplemented EBM2 culture medium. After four days, the cells were detached with 1 mL of trypsin EDTA (0.25%), washed with PBS, and incubated for 20 min at room temperature in dark conditions, with the fluorochrome-labeled antibody. Mouse monoclonal anti-CD14-Peridinin-Chlorophyll-protein (PerCP) (BioLegend, San Diego, CA, USA, cat. 301848, clone M5E2, dilution 1:20), anti-CD34-allophycocyanin (APC) (BioLegend, San Diego, CA, USA, cat. 343608, clone 561, dilution 1:40), and anti-VEGFR2/KDR-PE (BioLegend, San Diego, CA, USA, cat. 359904, clone 7D4-6, dilution 1:60). At the end of the incubation period, after the incubation period, we fixed the cells with 2% paraformaldehyde for 20 min. We then washed the cells with PBS/3% albumin and analyzed them on a BD FACSCalibur flow cytometer (BD Biosciences, San Jose, CA, USA). FACSCalibur cytometer (BD Biosciences, CA, USA). An SSC vs. FSC plot was utilized to construct a gate within the monocyte region (Supplementary Figure S1B). In a second dot plot, the CD14+ gate was plotted KDR vs. CD34+. The quadrant (Q)1 and Q2 represented the percentage of CD14+/KDR+ cell, whereas Q2 and Q3 represent the CD14+/CD34+ cells. The same gating method was used for MOMCs (Supplementary Figure S1C). Controls were singlets, non-stained cells, and FMO for each antibody. All analyses were done on a BD FACSCalibur flow cytometer (BD Biosciences, San Jose, CA, USA). We collected 5000 events per experiment for analysis using FlowJo software V10 (BD Biosciences, San Jose, CA, USA).

### 2.6. Statistical Analysis

Based on a previous study [31], a sample size calculation assuming 80% statistical power and a 95% confidence level indicated that 16 participants were required to detect significant differences in HDL size between study groups. All data were assessed for normality and kurtosis using the skewness-kurtosis test. Normally distributed variables are presented as mean ± standard deviation, and between-group comparisons were performed using Student’s *t*-test or one-way ANOVA with Bonferroni post hoc correction. Non-normally distributed variables are presented as median and interquartile range (IQR) and were analyzed using the Kruskal–Wallis test. Prevalence data are expressed as counts and percentages, and group comparisons were conducted using the chi-square test. A Pearson correlation assessed the relationship between HDL glycation products and the kinase domain receptor (KDR) as a marker of endothelial differentiation of MOMCs. Multivariate linear analysis was used to evaluate the association between HDL glycation status and CD14^+^/KDR^+^ phenotypic expression, adjusting for age, sex, and BMI or for HDL characteristics.

A *p*-value < 0.05 was considered significant, and STATA/IC 12.0 (Stata Corp LLC, College Station, TX, USA) was used for statistical analysis.

## 3. Results

Sixty individuals were recruited (20 with normoglycemia, 20 with prediabetes, and 20 with T2D). The isolated HDL amount was insufficient for glycation assays (two subjects with prediabetes and two with T2D) and for cell assays (two subjects with normoglycemia, three with prediabetes, and two from the T2D group).

There was no difference in age, gender proportion, total cholesterol, LDL-C, triglycerides, ApoA1, ApoB-100, AST, ALT, eGFR, and hsCRP (Table 1). As expected, body mass index, hemoglobin A1C, and fasting glucose values were significantly higher in the prediabetes and T2D groups compared to the normoglycemic group. In contrast, HDL-C levels were significantly lower in individuals with T2D.

Analysis of HDL subclasses showed that proportions of HDL2b and HDL2a subfractions were lower, while the HDL3c was higher in T2D individuals (Table 2, *p* < 0.01 for all). Consequently, the mean HDL particle diameter was significantly smaller in the T2D group compared to both the prediabetes and normoglycemic groups. Interestingly, abnormalities in HDL subclasses were increasing across the prediabetes and diabetes groups. Despite these differences, the macromolecular composition of HDL, including the percentage of free and esterified cholesterol, triglycerides, phospholipids, and protein content, was similar among groups (Table 2).

**Figure 1:**
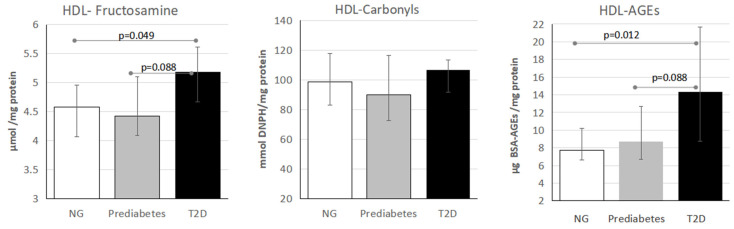
Glycation products in HDL from normoglycemic (NG), prediabetes, and type 2 diabetes (T2D) individuals. Fructosamine represents the early-, carbonyls the intermediate-, and AGEs the advanced-glycation products in HDL. *n* = 20 for each determination, except for AGEs (prediabetes *n* = 18 and T2D *n* = 18). *p* values were calculated by Kruskal–Wallis test.

Assessment of HDL glycation status showed that compared with normoglycemic and prediabetes individuals, those with T2D had higher fructosamine, early glycation, (4.6 [IQR: 4.0–4.9], 4.4 [IQR: 4.1–5.1], and 5.2 [IQR: 4.7–5.6] µmol/mg of protein, respectively; *p* = 0.049), and AGEs-HDL-(7.7 [IQR: 6.6–10.2], 8.7 [IQR: 6.7–12.7], and 14.3 [IQR: 8.7–21.7] µg BSA-AGEs/mg-HDL, respectively; *p* = 0.012). Nevertheless, the concentration of the intermediate glycation products (HDL-reactive carbonyls) was similar among groups (Figure 1).

To determine the optimal conditions for endothelial differentiation from fresh monocytes, cells were incubated with EBM2 medium. Supplementary Figure S1A shows that the maximum expression of KDR+ was reached on day 4. Moreover, CD14+ indicates that cultured cells remain viable until that day. Based on these findings, cells collected on day 4 were defined as monocyte-derived multipotential cells (MOMCs). Flow cytometry analysis revealed that, compared with fresh monocytes (Supplementary Figure S1B), MOMCs (Supplementary Figure S1C) exhibit increased cell size and granularity along with decreased expression of CD14+ (95.4% vs. 77.9%, respectively) but increased expression of CD34+ (6.7% vs. 13.0%, respectively) and KDR+ (17.1% vs. 22.0%, respectively). Additionally, an analysis of five independent assays showed that MOMCs had higher proportions of CD14^+^/KDR^+^ (24.3% vs. 50.2%; *p* = 0.019), CD14^+^/CD34^+^ (7.0% vs. 9.3%; *p* = 0.128), and CD14^+^/CD34^+^/KDR^+^ (3.6% vs. 8.1%; *p* = 0.002) cells compared with fresh monocytes (Supplementary Figure S1D).

**Figure 2:**
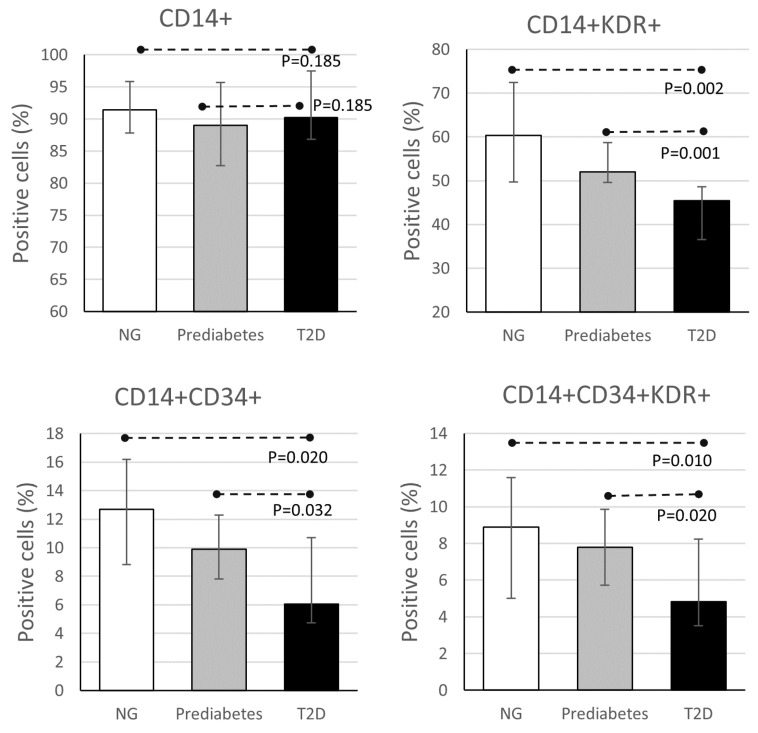
Expression of endothelial differentiation markers in MOMCs incubated with HDL of the studied groups. MOMCs were isolated as described in Methods Section and incubated twice with 50 μg of HDL for 45 min each 24 h, during 2 days. Then, the cells were maintained with EBM2 supplemented with vascular growth factors until harvested. NG = normoglycemic (*n* = 18); prediabetes (*n* = 17); and T2D = type 2 diabetes (*n* = 18). Kruskal–Wallis test was used to determine *p* values.

To assess the effect of HDL on the endothelial phenotype of MOMCs, cells were incubated with HDL isolated from individuals of the three groups. HDL from prediabetes and T2D groups induced an approximate 10% reduction in the proportion of CD14^+^ cells. Of note, HDL from prediabetic and T2D individuals caused a significant and progressive decrease in the proportions of CD14^+^/KDR^+^, CD14^+^/CD34^+^, and CD14^+^/CD34^+^/KDR^+^ cells (Figure 2).

Regarding HDL composition, pooled analysis (*n* = 52) revealed that HDL-triglyceride content was positively correlated with CD14^+^/KDR^+^ cell expression (r = 0.295, *p* = 0.034). In contrast, HDL-free cholesterol (r = −0.058, *p* = 0.685), esterified cholesterol (r = −0.255, *p* = 0.071), phospholipids (r = −0.191, *p* = 0.179), and protein (r = 0.112, *p* = 0.433) were not significantly associated with the endothelial phenotype.

**Figure 3:**
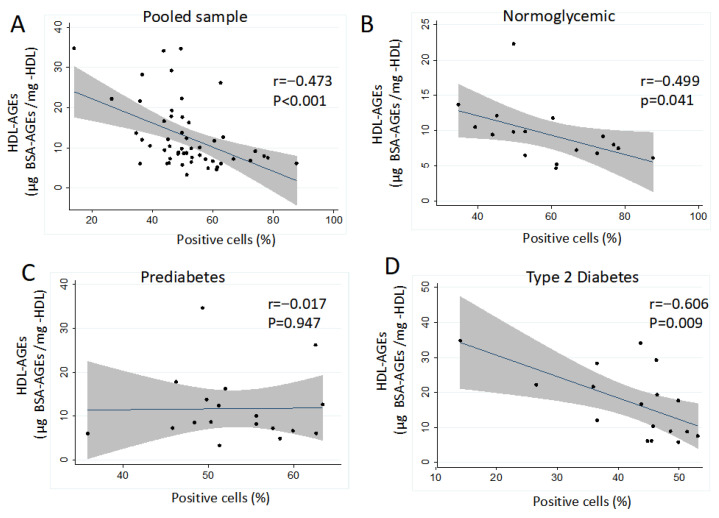
Correlation of HDL-AGEs with the percentage of CD14+KDR+ cells in individuals from the studied groups, Pooled sample, *n* = 52 (**A**); normoglycemic group, *n* = 18 (**B**); prediabetes group, *n* = 17 (**C**); and T2D patients, *n* = 17 (**D**). Pearson correlation coefficient (r) values.

While HDL-carbonyl content was not significantly associated with CD14^+^/KDR^+^ cell levels (r = −0.118, *p* = 0.405), both HDL-fructosamine (r = −0.289, *p* = 0.037) and HDL-AGEs (r = −0.473, *p* < 0.001) were inversely correlated with this endothelial marker (Figure 3A). This negative correlation was evident in the normoglycemic group (Figure 3B) and became more pronounced in individuals with T2D (Figure 3D). A multivariate linear analysis between HDL glycation status and CD14^+^/KDR^+^ phenotypic expression, showed that HDL-AGEs were associated independently of age, sex, and BMI, as well as HDL particle composition and the HDL mean diameter (Table 3).

## 4. Discussion

T2D is a highly prevalent metabolic disorder characterized by chronic hyperglycemia, increased oxidative stress, and accumulation of AGEs, all of which contribute to endothelial dysfunction, vascular injury, and atherogenesis [1,2]. HDL are macromolecular complexes that play a central role in reverse cholesterol transport, which could protect against the atherogenesis process [32]. Moreover, HDL contribute to preserving vascular integrity by promoting angiogenesis [14] through MOMCs differentiation along the endothelial lineage [11]. However, exposure to elevated glucose concentrations induces the formation of early and intermediate glycation adducts, which, although partially reversible, impair the antiatherogenic properties of HDL [3,16,17]. When hyperglycemia is sustained, the progressive accumulation of AGEs on HDL is favored [16]. Consequently, both the duration and intensity of hyperglycemia could determine the extent of HDL damage and, in turn, its capacity to exert vascular protective functions [18].

Despite these observations, the mechanisms underlying such impairment remain unclear, particularly concerning the role of the early glycation status of HDL in modulating the endothelial differentiation capacity of MOMCs. Results of the present study show that although HDL macromolecular composition was similar among normoglycemic, prediabetic, and T2D individuals, the size particles were smaller and enriched in early- and advanced-glycation products, among those with T2D. More interestingly, the research highlighted that HDL isolated from prediabetic and T2D individuals promotes a gradual decrease in CD14+/KDR+ expression, indicating a diminished endothelial phenotypic expression potential across the spectrum of HDL damage. This impairment was further confirmed by a significant inverse association between AGEs-HDL and cells expressing CD14+/KDR+ as endothelial markers. The findings suggest that glycemic control could be imperative in individuals, even at the early stages of glucose metabolism damage.

Endothelial cell dysfunction is common in high-glucose environments such as prediabetes or diabetes [19]. Consistently, clinical and experimental evidence has shown that vascular complications are the main cause of death in T2D patients. Although endothelial dysfunction has been linked to vascular damage in these patients, the early mechanisms that explain that association are not completely understood [1,20]. During the vascular homeostasis processes, several subpopulations of progenitor cells participate in the endothelial repair. MOMCs are a type of progenitor cells that exhibit remarkable plasticity, which contributes to vascular restoration through their differentiation into an endothelial cell-like phenotype, characterized by the expression of KDR while retaining the expression of CD14 and CD34 receptors [9,11,33,34]. Results of the present study contribute to knowing that HDL isolated from individuals with T2D diminished the endothelial phenotypic expression of endothelial precursor cells, evaluated through the expression of CD14+/KDR+ in MOMCs cultures (Figure 2). Further, the results highlighted that the CD14+/KDR+ expression negatively correlates with AGEs content in HDL from the sample as a whole, and that this correlation was stronger among T2D patients (Figure 3). The data suggests that HDL’s glycation status, which might start in prediabetic individuals [25], contributes to the lessened ability to restore vascular integrity. These findings are supported by reports showing that AGEs interfere with the ability of HDL to bind or signal through scavenger receptor class B type I (SRBI) [35,36]. Moreover, it has been previously reported that SRBI may regulate angiogenesis [37] and KDR expression [23]. On the other hand, there is evidence suggesting that AGEs receptor activation could downregulate the expression of ATP-binding cassette transporter A1 (ABCA1), and G1 (ABCG1), and SRBI receptors, which in turn may interfere with the beneficial effects of HDL on MOMCs [38,39].

Multiple in vivo and experimental studies have shown that HDL composition abnormalities affect endothelial functions. HDL abnormalities include phospholipid enrichment [40], depletion of sphingosine-1-phosphate (S1P) [41], and elevated triglyceride content [32], which increase susceptibility to oxidation, impair endothelial nitric oxide synthase (eNOS) activity, and compromise cholesterol efflux, respectively. Moreover, most of these abnormalities have been found to be associated with small HDL particle size [15]. Although the present study showed minimal alterations in most HDL components, we observed a significant correlation between MOMCs KDR expression and HDL triglycerides content. Interestingly, HDL particle size was not correlated with endothelial MOMCs differentiation. Despite glycation has been shown to accelerate apolipoprotein A1 catabolism [16], which could contribute to HDL size reduction in T2D, it is possible that the lack of association of HDL size and KDR could be explained by the clinical characteristics of our series, which include patients with a recent diagnosis of T2D. This might be supported by the fact that HDL-C levels gradually decreased among prediabetes and T2D individuals (Table 1). It is in line with the results of Al-Saudi et al. [42], who demonstrated that HDL isolated from individuals with prediabetes and T2D exhibited a gradual higher concentration of glycation products. This supports the notion that enhanced glycation is a progressive process, initiating as early as the prediabetic stage [20].

### Strengths and Limitations

One of the main strengths of this study is the comprehensive characterization of the individuals, which minimizes potential biases derived from comorbidities and associated treatments that are common in T2D patients, principally among patients with a long evolution time. This analysis allows us to use HDL characteristics as indicators of metabolic alterations, shedding light on their impact on vascular impairment in T2D. However, it is essential to acknowledge the study limitations, which include the relatively small sample size and the cross-sectional design. The latter does not allow for the establishment of causality. Therefore, correlation findings should be interpreted with caution due to their limited statistical power and validated through larger longitudinal studies. Additionally, the present study did not include assessments of endothelial functionality in MOMCs, such as endothelial nitric oxide synthase expression or tube formation assays. It should be noted that assessing HDL and their structural characteristics involves time-consuming techniques. Finally, the present study did not consider other AGEs such as methylglyoxal or glycol-aldehyde. Instead, our focus was on determining carboxymethyl-lysine and pentosidine species. It is essential to underline that these products are known to be present in HDL proteins, with apolipoprotein A1 constituting approximately 80% of the protein component. These findings underscore the need to develop and standardize glycation measurement techniques that can offer insights beyond conventional glycemic control measures.

## 5. Conclusions

HDL from newly diagnosed T2D individuals have higher glycation levels, negatively impacting KDR expression in MOMCs and reducing endothelial differentiation capacity. The results also highlight the significance of glycemic control during the prediabetes stages, where vascular damage is initiated due to compromised HDL composition and function. These results emphasize the importance of early interventions to preserve HDL integrity, preserve endothelial health, and prevent vascular complications in prediabetic and T2D individuals.

## Figures and Tables

**Table 1:** Clinical and metabolic characteristics of the studied groups.

	Normoglycemic *n* = 20	Prediabetes *n* = 20	Type 2 Diabetes *n* = 20	*p* Trend ^a^
Age (years)	49 ± 6.5	52 ± 5.9	51 ± 5.3	0.187
Gender (male/female)	7/13	7/13	10/10	0.535
BMI (kg/m^2^)	25.3 ± 3.3	28.1 ± 4.1 *	27.9 ± 3.9	0.039
Smoking, *n* (%)	3 (15)	6 (30)	2 (10)	0.320
Hemoglobin A1C (%)	5.5 (5.3–5.6)	5.9 (5.8–6.1) *	6.6 (6.5–7.4) *^†^	<0.001
Fasting glucose (mg/dL)	89 (83–95)	100 (96–105) *	126 (112–148) *^†^	<0.001
Total cholesterol (mg/dL)	194 (167–220)	203 (178–217)	171 (157–208)	0.260
LDL cholesterol (mg/dL)	122 (95–142)	127 (115–149)	108 (91–137)	0.250
HDL cholesterol (mg/dL)	51.5 (39–60)	44.4 (41–59)	38.2 (31–48) *^†^	0.019
Triglycerides (mg/dL)	108 (82–150)	115 (102–171)	148 (123–181)	0.158
ApoA1 (mg/dL)	151 ± 29	141 ± 19	139 ± 27	0.376
ApoB-100 (mg/dL)	109 ± 27	115 ± 29	115 ± 26	0.608
AST (IU/L)	20 (18–24)	18.4 (16–23)	22 (17–25)	0.457
ALT (IU/L)	22 (14–37)	19 (14–29)	25 (19–36)	0.235
eGFR (mL*min/1.73 m^2^)	94 (85–104)	97 (87–103)	103 (84–109)	0.614
hsCRP (mg/L)	1.4 (0.8–2.2)	1.1 (0.61–2.8)	1.9 (0.9–5.1)	0.320

Values are expressed as mean ± standard deviation, median (interquartile range) or number of subjects (%). BMI: body mass index; HDL cholesterol: High-density lipoprotein cholesterol; LDL cholesterol: low-density lipoprotein cholesterol; Apo: apolipoprotein; AST: aspartate aminotransferase; ALT: alanine aminotransferase. eGFR: estimated glomerular filtration rate; hsCRP: high-sensitive C-reactive protein. * *p* < 0.05 vs. normoglycemic, ^†^ *p* < 0.05 vs. prediabetes. ^a^ The *p*-value was calculated using one-way ANOVA, Kruskal–Wallis or chi-square test, as appropriate.

**Table 2:** Subfractions, size and composition of HDL particles in the studied groups.

	Normoglycemic *n* = 20	Prediabetes *n* = 20	Type 2 Diabetes *n* = 20	*p* Trend
HDL2b (%)	8.8 ± 3.9	7.83 ± 2.2	6.28 ± 2.6 *^†^	0.018
HDL2a (%)	17.5 ± 3.7	15.4 ± 3.8	13.4 ± 4.9 *^†^	0.001
HDL3a (%)	23.2 ± 2.7	23.4 ± 2.6	21.5 ± 4.2	0.105
HDL3b (%)	25.4 ± 2.7	26.4 ± 2.6	25.9 ± 2.6	0.430
HDL3c (%)	24 ± 6.6	27.0 ± 5.7	32.6 ± 10 *^†^	0.010
HDL mean diameter (nm)	8.51 ± 0.2	8.44 ± 0.2	8.32 ± 0.22 *^†^	0.002
HDL macromolecular composition				
Free cholesterol (%)	1.96 (1.72–2.22)	1.74 (1.22–2.32)	1.79 (1.23–2.21)	0.598
Esterified cholesterol (%)	20.1 (18–22)	20.7 (19–21)	20.6 (19–21)	0.964
Phospholipids (%)	3.8 (3.1–4.4)	3.4 (2.9–4.5)	4.1 (2.9–5.9)	0.502
Triglycerides (%)	21.7 (20–22)	21.6 (20–22)	21.5 (20–22)	0.866
Protein (%)	51.8 (50–53)	52.7 (50–54)	51.3 (48–55)	0.778

Values are expressed as mean ± standard deviation or median (interquartile range). * *p* < 0.05 vs. normoglycemic, ^†^ *p* < 0.05 vs. prediabetes.

**Table 3:** Multivariate linear analysis between HDL glycation and CD14^+^/KDR^+^ phenotypic expression.

	Univariate (Beta Coefficient)	*p* Value	Adjusted for Age, Sex and BMI (Beta Coefficient)	*p* Value	Multivariate (Beta Coefficient)	*p* Value
HDL-Fructusamine (µmol/mg of protein)	−0.289	0.038	−0.277	0.052	−0.265	0.096
HDL-Carbonyls (mmol DNPH/mg protein)	−0.118	0.405	−0.147	0.324	−0.070	0.612
HDL-AGEs (µg-BSA-AGEs/mg protein)	−0.473	<0.001	−0.454	0.002	−0.418	0.002

The adjusted model included the HDL mean diameter and the macromolecular composition of HDL: free cholesterol, esterified cholesterol, phospholipids, triglycerides, and protein. BMI: Body mass index.

## Data Availability

The data supporting the findings of this study are accessible upon reasonable request from the corresponding author.

## Supplementary Materials

The following supporting information can be downloaded at: https://www.mdpi.com/article/10.3390/metabo16030194/s1, Figure S1: Comparative CD14+, KDR+, and CD34+ expression in fresh monocytes and MOMCs from healthy individuals. CD14+ and KD+R expression in fresh monocytes cultivated with EBM2 medium throughout 14 days (A); comparison of size (FSC), granularity (SCC), and expression of CD14+, CD34+ and KDR+ on fresh monocytes (B) and MOMCs (C) cultivated for 4 days; average percentage (*n* = 5) of cells expressing CD14+/KDR+, CD14+/CD34+, or CD14+/CD34+//KDR+, in fresh monocytes and MOMCs (D). Flow cytometry colors show unlabeled cells (red) or labeled cells (blue), with antibodies coupled to fluorophores (CD14-PerCP, CD34-APC and KDR-PE). Statistical analysis was performed using a Student *t*-test; * *p* < 0.05.

## Abbreviations

AGEs	advanced glycation end products
APC	allophycocyanin
EBM2	endothelial cell growth basal medium-2
EPC	endothelial progenitor cells
HDL	High-density lipoproteins
MOMCs	Monocyte-derived multipotential cells
PE	phycoerythrin
PerCP	Peridinin-chlorophyll-protein
LDL	low-density lipoprotein cholesterol
T2D	Type 2 diabetes
VEGFR2/KDR	vascular endothelial grown factor receptor 2/kinase domain receptor
SRB-I	scavenger receptor class B type I

## References

[1] DeFronzo R.A. (2010). Insulin Resistance, Lipotoxicity, Type 2 Diabetes and Atherosclerosis: The Missing Links. The Claude Bernard Lecture 2009. Diabetologia.

[2] Henning R.J. (2018). Type-2 Diabetes Mellitus and Cardiovascular Disease. Future Cardiol..

[3] Twarda-Clapa A., Olczak A., Białkowska A.M., Koziołkiewicz M. (2022). Advanced Glycation End-Products (AGEs): Formation, Chemistry, Classification, Receptors, and Diseases Related to AGEs. Cells.

[4] Medina R.J., Barber C.L., Sabatier F., Dignat-George F., Melero-Martin J.M., Khosrotehrani K., Ohneda O., Randi A.M., Chan J.K.Y., Yamaguchi T. (2017). Endothelial Progenitors: A Consensus Statement on Nomenclature. Stem Cells Transl. Med..

[5] Medina R.J., O’Neill C.L., O’Doherty T.M., Knott H., Guduric-Fuchs J., Gardiner T.A., Stitt A.W. (2011). Myeloid Angiogenic Cells Act as Alternative M2 Macrophages and Modulate Angiogenesis through Interleukin-8. Mol. Med..

[6] Nasiri B., Yi T., Wu Y., Smith R.J., Podder A.K., Breuer C.K., Andreadis S.T. (2022). Monocyte Recruitment for Vascular Tissue Regeneration. Adv. Healthc. Mater..

[7] Smith R.J., Nasiri B., Kann J., Yergeau D., Bard J.E., Swartz D.D., Andreadis S.T. (2020). Endothelialization of Arterial Vascular Grafts by Circulating Monocytes. Nat. Commun..

[8] Seta N., Kuwana M. (2010). Derivation of Multipotent Progenitors from Human Circulating CD14^+^ Monocytes. Exp. Hematol..

[9] Kuwana M., Okazaki Y., Kodama H., Izumi K., Yasuoka H., Ogawa Y., Kawakami Y., Ikeda Y. (2003). Human Circulating CD14^+^ Monocytes as a Source of Progenitors That Exhibit Mesenchymal Cell Differentiation. J. Leukoc. Biol..

[10] Möbius-Winkler S., Höllriegel R., Schuler G., Adams V. (2009). Endothelial Progenitor Cells: Implications for Cardiovascular Disease. Cytom. Part A.

[11] Kuwana M., Okazaki Y., Kodama H., Satoh T., Kawakami Y., Ikeda Y. (2006). Endothelial Differentiation Potential of Human Monocyte-Derived Multipotential Cells. Stem Cells.

[12] Lucchesi D., Popa S.G., Sancho V., Giusti L., Garofolo M., Daniele G., Pucci L., Miccoli R., Penno G., Del Prato S. (2018). Influence of High Density Lipoprotein Cholesterol Levels on Circulating Monocytic Angiogenic Cells Functions in Individuals with Type 2 Diabetes Mellitus. Cardiovasc. Diabetol..

[13] Jin F., Hagemann N., Sun L., Wu J., Doeppner T.R., Dai Y., Hermann D.M. (2018). High-Density Lipoprotein (HDL) Promotes Angiogenesis via S1P3-Dependent VEGFR2 Activation. Angiogenesis.

[14] Primer K.R., Psaltis P.J., Tan J.T.M., Bursill C.A. (2020). The Role of High-Density Lipoproteins in Endothelial Cell Metabolism and Diabetes-Impaired Angiogenesis. Int. J. Mol. Sci..

[15] Rye K.-A., Barter P.J. (2014). Regulation of High-Density Lipoprotein Metabolism. Circ. Res..

[16] Kashyap S.R., Osme A., Ilchenko S., Golizeh M., Lee K., Wang S., Bena J., Previs S.F., Smith J.D., Kasumov T. (2018). Glycation Reduces the Stability of ApoAI and Increases HDL Dysfunction in Diet-Controlled Type 2 Diabetes. J. Clin. Endocrinol. Metab..

[17] Ravi R., Ragavachetty Nagaraj N., Subramaniam Rajesh B. (2020). Effect of Advanced Glycation End Product on Paraoxonase 2 Expression: Its Impact on Endoplasmic Reticulum Stress and Inflammation in HUVECs. Life Sci..

[18] Bansal S., Burman A., Tripathi A.K. (2023). Advanced Glycation End Products: Key Mediator and Therapeutic Target of Cardiovascular Complications in Diabetes. World J. Diabetes.

[19] Clyne A.M. (2021). Endothelial Response to Glucose: Dysfunction, Metabolism, and Transport. Biochem. Soc. Trans..

[20] Su Y., Liu X.-M., Sun Y.-M., Jin H.-B., Fu R., Wang Y.-Y., Wu Y., Luan Y. (2008). The Relationship between Endothelial Dysfunction and Oxidative Stress in Diabetes and Prediabetes. Int. J. Clin. Pract..

[21] Kang H., Ma X., Liu J., Fan Y., Deng X. (2017). High Glucose-Induced Endothelial Progenitor Cell Dysfunction. Diabetes Vasc. Dis. Res..

[22] Sorrentino S.A., Besler C., Rohrer L., Meyer M., Heinrich K., Bahlmann F.H., Mueller M., Horváth T., Doerries C., Heinemann M. (2010). Endothelial-Vasoprotective Effects of High-Density Lipoprotein Are Impaired in Patients with Type 2 Diabetes Mellitus but Are Improved after Extended-Release Niacin Therapy. Circulation.

[23] Li H.-M., Mo Z.-W., Peng Y.-M., Li Y., Dai W.-P., Yuan H.-Y., Chang F.-J., Wang T.-T., Wang M., Hu K.-H. (2020). Angiogenic and Antiangiogenic Mechanisms of High Density Lipoprotein from Healthy Subjects and Coronary Artery Diseases Patients. Redox Biol..

[24] Gomes Kjerulf D., Wang S., Omer M., Pathak A., Subramanian S., Han C.Y., Tang C., den Hartigh L.J., Shao B., Chait A. (2020). Glycation of HDL Blunts Its Anti-Inflammatory and Cholesterol Efflux Capacities in Vitro, but Has No Effect in Poorly Controlled Type 1 Diabetes Subjects. J. Diabetes Complicat..

[25] Jorge-Galarza E., Medina-Urrutia A., Posadas-Sánchez R., Posadas-Romero C., Cardoso-Saldaña G., Vargas-Alarcón G., Caracas-Portilla N., González-Salazar C., Torres-Tamayo M., Juárez-Rojas J.G. (2016). Adipose Tissue Dysfunction Increases Fatty Liver Association with Pre Diabetes and Newly Diagnosed Type 2 Diabetes Mellitus. Diabetol. Metab. Syndr..

[26] American Diabetes Association (2018). 2. Classification and Diagnosis of Diabetes: Standards of Medical Care in Diabetes-2018. Diabetes Care.

[27] DeLong D.M., DeLong E.R., Wood P.D., Lippel K., Rifkind B.M. (1986). A Comparison of Methods for the Estimation of Plasma Low- and Very Low-Density Lipoprotein Cholesterol. The Lipid Research Clinics Prevalence Study. JAMA.

[28] Medina-Urrutia A., Juarez-Rojas J.G., Martínez-Alvarado R., Jorge-Galarza E., Posadas-Sánchez R., Cardoso-Saldaña G., Caracas-Portilla N., Mendoza-Perez E., Posadas-Romero C. (2008). High-Density Lipoprotein Subclasses Distribution and Composition in Mexican Adolescents with Low HDL Cholesterol and/or High Triglyceride Concentrations, and Its Association with Insulin and C-Reactive Protein. Atherosclerosis.

[29] Blanche P.J., Gong E.L., Forte T.M., Nichols A.V. (1981). Characterization of Human High-Density Lipoproteins by Gradient Gel Electrophoresis. Biochim. Biophys. Acta.

[30] Oliver C.N., Ahn B.W., Moerman E.J., Goldstein S., Stadtman E.R. (1987). Age-Related Changes in Oxidized Proteins. J. Biol. Chem..

[31] Juárez-Rojas J., Medina-Urrutia A., Posadas-Sánchez R., Jorge-Galarza E., Mendoza-Pérez E., Caracas-Portilla N., Cardoso-Saldaña G., Muñoz-Gallegos G., Posadas-Romero C. (2008). High-Density Lipoproteins Are Abnormal in Young Women with Uncomplicated Systemic Lupus Erythematosus. Lupus.

[32] Skeggs J.W., Morton R.E. (2002). LDL and HDL Enriched in Triglyceride Promote Abnormal Cholesterol Transport. J. Lipid Res..

[33] Hur J., Yoon C.-H., Kim H.-S., Choi J.-H., Kang H.-J., Hwang K.-K., Oh B.-H., Lee M.-M., Park Y.-B. (2004). Characterization of Two Types of Endothelial Progenitor Cells and Their Different Contributions to Neovasculogenesis. Arterioscler. Thromb. Vasc. Biol..

[34] Chopra H., Hung M.K., Kwong D.L., Zhang C.F., Pow E.H.N. (2018). Insights into Endothelial Progenitor Cells: Origin, Classification, Potentials, and Prospects. Stem Cells Int..

[35] Ohgami N., Nagai R., Miyazaki A., Ikemoto M., Arai H., Horiuchi S., Nakayama H. (2001). Scavenger Receptor Class B Type I-Mediated Reverse Cholesterol Transport Is Inhibited by Advanced Glycation End Products. J. Biol. Chem..

[36] Miyazaki A., Nakayama H., Horiuchi S. (2002). Scavenger Receptors That Recognize Advanced Glycation End Products. Trends Cardiovasc. Med..

[37] Kratzer A., Giral H., Landmesser U. (2014). High-Density Lipoproteins as Modulators of Endothelial Cell Functions: Alterations in Patients with Coronary Artery Disease. Cardiovasc. Res..

[38] Daffu G., Shen X., Senatus L., Thiagarajan D., Abedini A., Hurtado Del Pozo C., Rosario R., Song F., Friedman R.A., Ramasamy R. (2015). RAGE Suppresses ABCG1-Mediated Macrophage Cholesterol Efflux in Diabetes. Diabetes.

[39] Nagata H., Lyu J., Imachi H., Fukunaga K., Sato S., Kobayashi T., Saheki T., Seo K., Salimah J.B., Iwama H. (2021). AGEs Inhibit Scavenger Receptor Class B Type I Gene Expression via Smad1 in HUVECs. J. Mol. Endocrinol..

[40] Navab M., Ananthramaiah G.M., Reddy S.T., Van Lenten B.J., Ansell B.J., Fonarow G.C., Vahabzadeh K., Hama S., Hough G., Kamranpour N. (2004). The Oxidation Hypothesis of Atherogenesis: The Role of Oxidized Phospholipids and HDL. J. Lipid Res..

[41] Denimal D., Monier S., Brindisi M.-C., Petit J.-M., Bouillet B., Nguyen A., Demizieux L., Simoneau I., Pais de Barros J.-P., Vergès B. (2017). Impairment of the Ability of HDL from Patients with Metabolic Syndrome but without Diabetes Mellitus to Activate eNOS: Correction by S1P Enrichment. Arterioscler. Thromb. Vasc. Biol..

[42] Al Saudi R.M., Kasabri V., Naffa R., Bulatova N., Bustanji Y. (2018). Glycated LDL-C and Glycated HDL-C in Association with Adiposity, Blood and Atherogenicity Indices in Metabolic Syndrome Patients with and without Prediabetes. Ther. Adv. Endocrinol. Metab..

