# Supplementary figures and images for: Glycated High-Density Lipoproteins Reduce Endothelial Phenotypic Expression of Monocyte-Derived Multipotential Cells in Early Type 2 Diabetes
Source: Metabolites. 2026 Mar 15;16(3):194. doi: 10.3390/metabo16030194 (PMC13027787; doi:10.3390/metabo16030194)

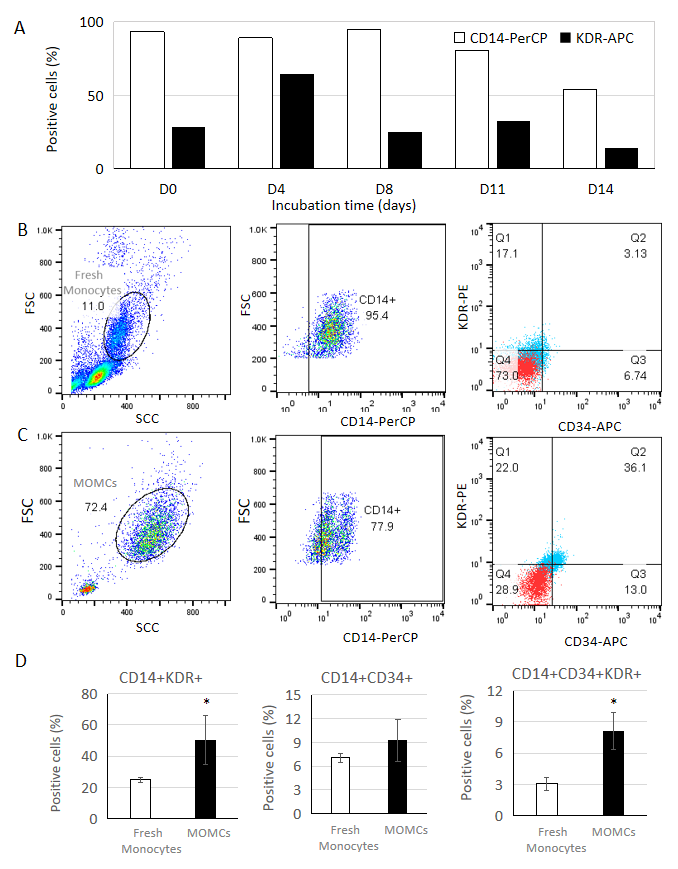

Supplement: Supplementary file 1 [file metabolites-16-00194-s001.zip › metabolites-4140329-supplementary.png]
